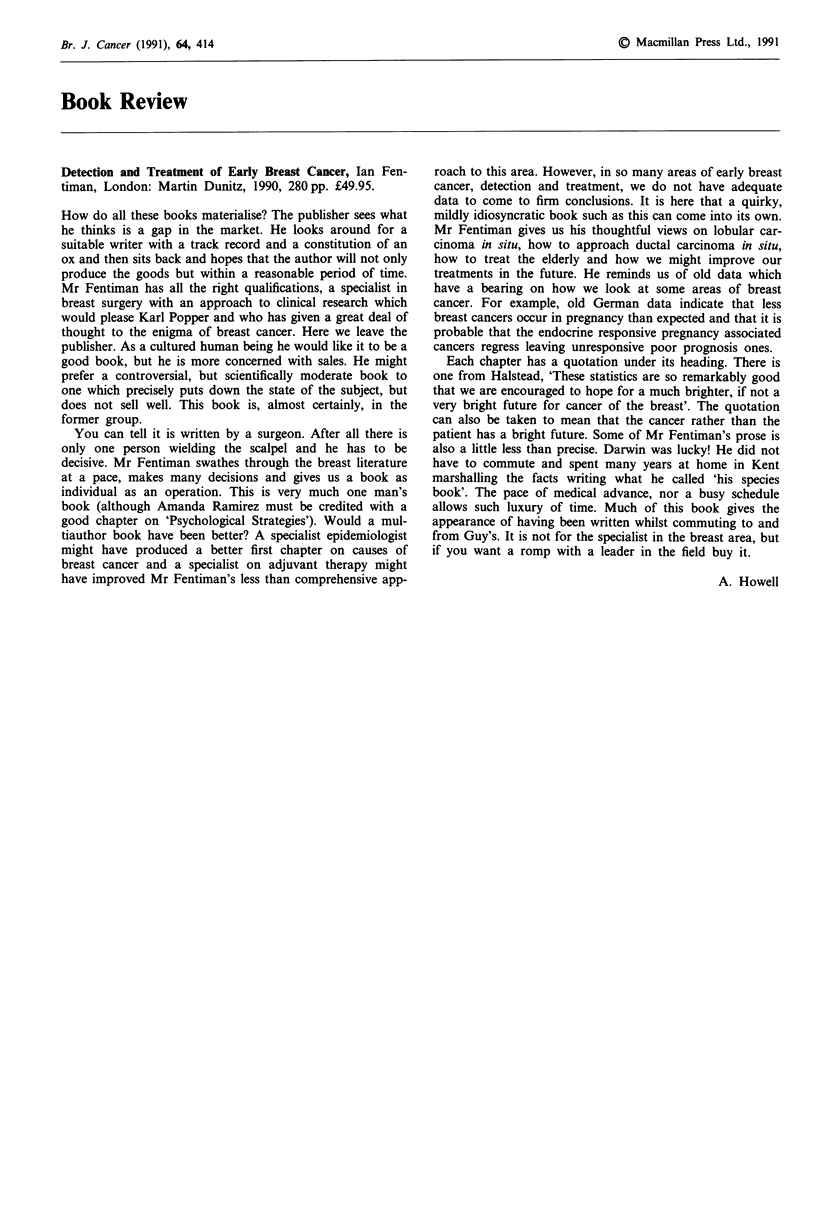# Detection and Treatment of Early Breast Cancer

**Published:** 1991-08

**Authors:** A. Howell


 © Macmillan Press Ltd., 1991

### Book Review

**Detection and Treatment of Early Breast Cancer,** Ian Fentiman, London: Martin Dunitz, 1990, 280 pp. £49.95.

How do all these books materialise? The publisher sees what he thinks is a gap in the market. He looks around for a suitable writer with a track record and a constitution of an ox and then sits back and hopes that the author will not only produce the goods but within a reasonable period of time. Mr Fentiman has all the right qualifications, a specialist in breast surgery with an approach to clinical research which would please Karl Popper and who has given a great deal of thought to the enigma of breast cancer. Here we leave the publisher. As a cultured human being he would like it to be a good book, but he is more concerned with sales. He might prefer a controversial, but scientifically moderate book to one which precisely puts down the state of the subject, but does not sell well. This book is, almost certainly, in the former group.

You can tell it is written by a surgeon. After all there is only one person wielding the scalpel and he has to be decisive. Mr Fentiman swathes through the breast literature at a pace, makes many decisions and gives us a book as individual as an operation. This is very much one man's book (although Amanda Ramirez must be credited with a good chapter on 'Psychological Strategies'). Would a multiauthor book have been better? A specialist epidemiologist might have produced a better first chapter on causes of breast cancer and a specialist on adjuvant therapy might have improved Mr Fentiman's less than comprehensive approach to this area. However, in so many areas of early breast cancer, detection and treatment, we do not have adequate data to come to firm conclusions. It is here that a quirky, mildly idiosyncratic book such as this can come into its own. Mr Fentiman gives us his thoughtful views on lobular carcinoma *in situ*, how to approach ductal carcinoma *in situ*, how to treat the elderly and how we might improve our treatments in the future. He reminds us of old data which have a bearing on how we look at some areas of breast cancer. For example, old German data indicate that less breast cancers occur in pregnancy than expected and that it is probable that the endocrine responsive pregnancy associated cancers regress leaving unresponsive poor prognosis ones.

Each chapter has a quotation under its heading. There is one from Halstead, 'These statistics are so remarkably good that we are encouraged to hope for a much brighter, if not a very bright future for cancer of the breast'. The quotation can also be taken to mean that the cancer rather than the patient has a bright future. Some of Mr Fentiman's prose is also a little less than precise. Darwin was lucky! He did not have to commute and spent many years at home in Kent marshalling the facts writing what he called 'his species book'. The pace of medical advance, nor a busy schedule allows such luxury of time. Much of this book gives the appearance of having been written whilst commuting to and from Guy's. It is not for the specialist in the breast area, but if you want a romp with a leader in the field buy it.

A. Howell